# Special Issue “Retinal Diseases and Macular Degeneration: Cell Biology and Molecular Genetics”

**DOI:** 10.3390/ijms27094022

**Published:** 2026-04-30

**Authors:** Simona Alibrandi, Luigi Donato

**Affiliations:** 1 Department of Biomedical and Dental Sciences and Morphofunctional Imaging, University of Messina, Via Consolare Valeria 1, 98125 Messina, Italy; simona.alibrandi@unime.it; 2 Department of Biomolecular Strategies, Genetics, Cutting-Edge Therapies, I.E.ME.S.T., Via Michele Miraglia 20, 90139 Palermo, Italy

Retinal diseases and macular degeneration continue to represent major global health challenges, having profound personal, social, and economic implications. As life expectancy increases worldwide, the prevalence of age-related retinal disorders is projected to substantially grow. Inherited retinal dystrophies—once considered rare—are now diagnosed with increasing accuracy owing to advances in genetics and multimodal imaging. However, despite remarkable scientific progress, many of these diseases remain incompletely treatable or, in several cases, untreatable. Deeper mechanistic insight and innovative therapeutic strategies are thus urgently needed.

Within this context, we present this Special Issue *“Retinal Diseases and Macular Degeneration: Cell Biology and Molecular Genetics”*, which gathers six articles that span the spectrum of retinal research from molecular genetics to developmental biology, advanced drug-delivery technologies, and translational neuroimmunology. Although diverse in focus, these studies converge on shared themes: the complexity of retinal homeostasis, the power of novel genomic tools, and the emergence of new therapeutics capable of reshaping the future of retinal care. This collection underscores the strength of interdisciplinarity and the importance of connecting basic science with clinical applicability.

One of the central challenges in treating and managing retinal disease is the persistence of vision-threatening complications despite surgical and technological advances. This challenge is illustrated by a review focused on proliferative vitreoretinopathy (PVR), a major cause of failed retinal detachment repair (Contribution 1). Although vitreoretinal surgery continues to evolve, the outcomes remain suboptimal, mainly because PVR is driven by complex fibroinflammatory pathways and multicellular responses that surgical approaches alone cannot resolve. Contribution 1 highlights the promise of nanotechnology-based drug delivery systems—including nanoparticles, liposomes, and dendrimers—to target early profibrotic signals, overcome ocular barriers, and reduce postoperative complications. The analysis clarifies the pathogenic mechanisms of PVR and envisions a future where mini-invasive nanotherapies complement or even replace aspects of surgical intervention.

Genetic research is another focus of this Special Issue. Whole-genome sequencing (WGS) is rapidly redefining the diagnosis of inherited retinal diseases, especially in cases where conventional panels or whole-exome sequencing (WES) fail to capture critical structural variants. Contribution 2 demonstrates this change in diagnosing inherited retinal diseases through identifying two pathogenic BBS5 variants, such as a large exon-level deletion missed by multiple NGS platforms, in a patient with Bardet–Biedl syndrome (Contribution 2). By combining advanced sequencing with functional assays assessing ciliary morphology and Sonic Hedgehog signaling, the study emphasizes the importance of WGS for resolving complex cases and cautions against relying solely on standard exome-based diagnostics. The findings broaden the spectrum of known BBS5 mutations and highlight the essential role of ciliary integrity in retinal and systemic diseases.

The growing availability of high-throughput sequencing is transforming the clinical characterization of retinitis pigmentosa (RP), the most common inherited retinal dystrophy. A comprehensive cohort study integrating genetic testing, optical coherence tomography (OCT), and fundus autofluorescence (FAF) delineated phenotype–genotype correlations in nearly 200 patients (Contribution 3). Diagnostic variants were identified in over half of the cases, illustrating the power and the remaining limitations of the current gene panels. The analysis of ellipsoid zone width and FAF patterns provides insights into disease staging and potential eligibility for emerging gene-based therapies. The authors also underscore the heterogeneity of RP and the need for continued expansion of genetic reference databases to better serve diverse populations.

Contribution 4 complementarily extends this multimodal and genetic approach to the severe end of the inherited retinal disease spectrum: Leber congenital amaurosis (LCA) and early-onset severe retinal dystrophies. A study of 34 patients revealed high rates of identifiable pathogenic variants, particularly in *CEP290*, *GUCY2D*, and *CRB*. The authors describe structural retinal changes detectable via OCT and FAF that are crucial for early diagnosis and therapeutic planning. Notably, real-world cases of patients qualifying for gene replacement therapy in RPE65-related disease are documented, illustrating how molecular diagnosis directly impacts clinical decision-making in the era of precision ophthalmology.

Beyond human genetics and clinical characterization, this Special Issue also explores the fundamental mechanisms through which the retina develops. Researchers investigated the role of protein kinase N1 (PKN1) in shaping the balance of retinal neuron populations, focusing on its interaction with the transcription factor NeuroD2 (Contribution 5). Using Pkn1 knockout models, the authors found that PKN1 acts as a regulator of NeuroD2 expression, influencing the development of amacrine cells and specific subtypes of retinal ganglion cells (α-RGCs). NeuroD2+ and Dab1+ amacrine populations are increased in Pkn1−/− retinae, with a selective reduction in α-RGCs, suggesting a nuanced developmental role that can inform future regenerative approaches or cell-type reprogramming strategies.

Finally, the vulnerability of the retina to neuroinflammation—a key driver of glaucomatous damage and other optic neuropathies—was addressed in a study centered on the P2X7 receptor, a mediator of ATP-induced inflammatory signaling (Contribution 6). The study shows that P2X7 activation is necessary and sufficient to induce complex patterns of astrocyte reactivity, including the pan-reactive, A1-type (neurotoxic), and A2-type (neuroprotective) signatures. The experiments demonstrate that the pharmacological stimulation of P2X7 increases the expression of upstream inflammatory mediators such as TNF-α, IL-1α, and C1qa, whereas the genetic deletion of P2X7 prevents the astrocytic activation typically observed after intraocular pressure elevation. These findings position P2X7 as a crucial node in retinal inflammatory cascades and highlight its potential as a therapeutic target in glaucoma.

Together, the contributions in this Special Issue offer an integrative perspective on retinal disease biology spanning genetic architecture, molecular signaling, developmental pathways, inflammatory responses, and emerging therapeutic innovations. Several cross-cutting themes emerge: the growing diagnostic power of WGS, the value of multimodal imaging as an objective biomarker of disease progression, the potential of nanotechnology and targeted drug delivery, and the central role of cellular diversity in shaping retinal vulnerability and resilience. These diverse approaches collectively describe pathways that may allow clinicians to not only halt retinal degeneration but also restore function and preserve sight in conditions currently deemed irreversible.

Looking ahead, the convergence of omics technologies, computational biology, regenerative engineering, and gene-based therapeutics promises to transform our understanding of retinal disorders. However, the success of these innovations will depend on sustained interdisciplinary collaboration connecting laboratory discoveries to clinical practice and ensuring equitable access to advanced diagnostics and therapies.

We express our deepest gratitude to all authors, reviewers, and colleagues whose work has made this Special Issue possible. Their contributions enrich the field and provide a foundation upon which future advances in retinal and macular disease research will grow. We hope this collection inspires continued investigation and scientific creativity toward a future with a diminished retinal disease burden.

